# Cestode infections in non-human primates suggest the existence of zoonotic cycles in the area surrounding the Strasbourg primatology center

**DOI:** 10.1051/parasite/2019025

**Published:** 2019-05-01

**Authors:** Valentin Greigert, Nicolas Brion, Cécile Lang, Pierrick Regnard, Alexander W. Pfaff, Ahmed Abou-Bacar, Fanélie Wanert, Manon Dirheimer, Ermanno Candolfi, Julie Brunet

**Affiliations:** 1 Unité d’infectiologie, Service de médecine interne, Hôpitaux Civils de Colmar 68000 Colmar France; 2 Institut de Parasitologie et de Pathologie Tropicale, EA 7292, Fédération de Médecine Translationnelle, Université de Strasbourg 67000 Strasbourg France; 3 École Vétérinaire d’Alfort 94700 Maisons-Alfort France; 4 Laboratoire de Parasitologie et Mycologie Médicales, Hôpitaux Universitaires de Strasbourg 67000 Strasbourg France; 5 Centre de Primatologie – SILABE (Simian Laboratory Europe) ADUEIS, Fort Foch 67205 Oberhausbergen France

**Keywords:** Echinococcosis, France, Primates, Public health, Taenia, Zoonosis

## Abstract

*Background*: Several cases of infections due to *Echinococcus multilocularis*, *Taenia martis* and *Taenia crassiceps* were recently described in various species of captive non-human primates (NHPs) harbored in the Strasbourg Primate Center (SPC). Furthermore, one of the first cases of human cysticercosis due to *T. martis* was described in the Strasbourg region. These data suggest the existence of zoonotic cycles of tapeworm infections in the direct environment of the SPC. The aim of our study was to assess the prevalence of larval cestode infections among intermediate and definitive hosts in the close neighborhood of the center. We analyzed carnivore mammal fecal samples as well as rodent carcasses, collected inside or near the SPC, using PCR. Furthermore, we performed serology for *Echinococcus* spp. and *Taenia* spp. on NHP sera. *Results*: We found that 14.5% (95% CI [8.6; 20.4]) of 138 carnivore feces were positive for *E. multilocularis*-DNA, as well as 25% (95% CI [5.5; 57.2]) of 12 rodent carcasses, and 5.1% (95% CI [1.4; 8.7]) for *T. martis* or *T. crassiceps*. Of all NHPs tested, 10.1% (95% CI [3.8; 16.4]) were seropositive for *Echinococcus* spp. and 8.2% (95% CI [1.3; 15.1]) for *Taenia* spp. *Conclusions*: Our data support the existence of zoonotic cycles of larval cestode infections in the direct environment of the primatology center affecting NHPs harbored in the SPC, potentially threatening the human population living in this area. Since this zoonotic risk is borne by local wildlife, and given the severity of these infections, it seems necessary to put in place measures to protect captive NHPs, and further studies to better assess the risk to human populations.

## Abstract (fr)

*Contexte* : Plusieurs cas de cestodoses larvaires dues à *Echinococcus multilocularis*, *Taenia martis* et *T. crassiceps* ont été récemment décrits chez des primates non-humains (PNH) captifs appartenant à diverses espèces, hébergés au Centre de Primatologie de Strasbourg (CdP). De plus, un des premiers cas humains de cysticercose due à *T. martis* a été décrit dans la région de Strasbourg. Ces données suggèrent l’émergence d’un nouveau foyer parasitaire dans l’environnement direct du CdP. Le but de notre étude était d’évaluer la prévalence des cestodoses larvaires chez les hôtes intermédiaires et définitifs de ces parasites dans le proche voisinage du CdP. Nous avons analysé des échantillons de selles de mammifères carnivores, ainsi que des carcasses de rongeurs, collectés à l’intérieur ou aux alentours du CdP. De plus, nous avons réalisé des sérologies pour *Echinococcus* spp. et *Taenia* spp. sur des sérums de PNH. *Résultats* : Nous avons trouvé que 14,5 % (IC95 % [8,6 ; 20,4]) des 138 selles de carnivores étaient positives pour *E. multilocularis*, ainsi que 25 % (IC95 % [5,5 ; 57,2]) des 12 carcasses de rongeur, et 5,1 % (IC95 % [1,4 ; 8,7]) pour *T. martis* ou *T. crassiceps*. De tous les PNH testés, 10,1 % (IC95 % [3,8 ; 16,4]) étaient positifs pour *Echinococcus* spp. et 8,2 % (IC95 % [1,3 ; 15,1]) pour *Taenia* spp. *Conclusions* : Nos données suggèrent l’existence de cycles zoonotiques de cestodoses larvaires dans l’environnement direct du centre de primatologie, affectant les PNH hébergés au CdP et menaçant potentiellement les populations humaines vivant dans cette zone. Ce risque zoonotique étant porté par la faune sauvage locale, et comptes tenus de la sévérité de ces infections, il semble nécessaire de mettre en place des mesures afin de protéger les PNH captifs, et de plus larges études afin d’évaluer le risque pour les populations humaines environnantes.

## Introduction

**Figure 1:**
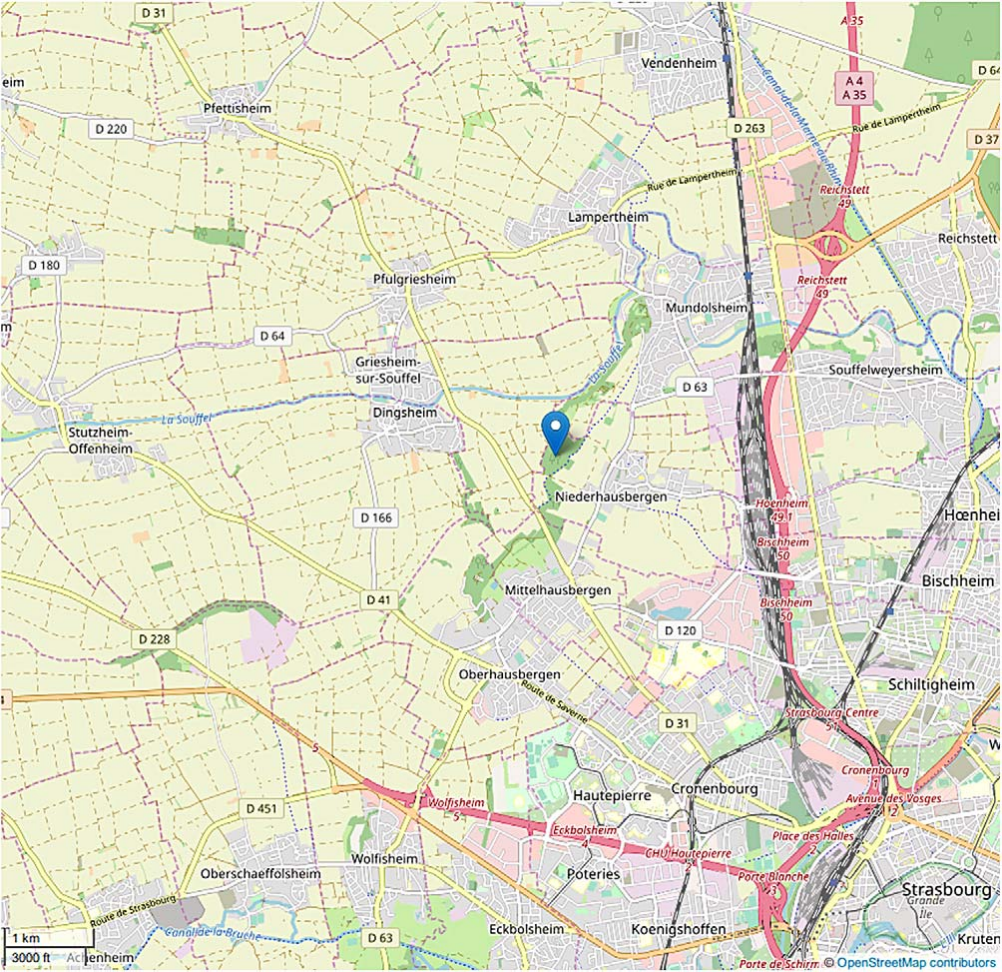
Location of the Strasbourg Primatology Center, close to the Strasbourg metropolitan area. The center is marked. From OpenStreetMap contributors, licensed as CC BY-SA, https://www.openstreetmap.org/copyright.

**Figure 2:**
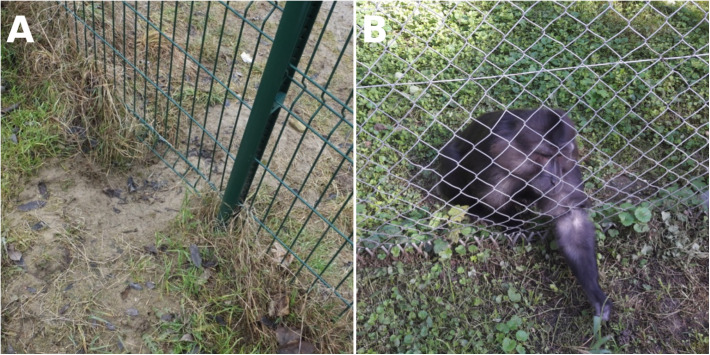
Two types of interaction between non-human primates and local wildlife. (A) Foxes (*Vulpes vulpes*) are able to dig passages under the fences of cages, and (B) non-human primates commonly look for food outside the fence, like this Tonkean macaque (*Macaca tonkeana*).

The Strasbourg Primate Center (SPC) is a scientific research center situated on the outskirts of the Strasbourg metropolitan area (Fig. 1). In this facility, many non-human primates (NHPs) belonging to various species live in semi-freedom in parks or large cages, allowing them to interact with local wildlife, including birds, rodents, and carnivore mammals such as red foxes (*Vulpes vulpes*) or pine martens (*Martes martes*) (Fig. 2). *Echinococcus* spp. and *Taenia* spp. are common cestodes in Eastern France, with a heteroxenous life cycle involving different wild species, carnivores as definitive hosts, and rodents as intermediate ones. Some of these parasites are known for being able to infect NHPs belonging to several species including gorillas, lemurs and macaques [5, 6, 8, 11, 26, 30, 35]. Indeed, between 2009 and 2018, five cases of infections in NHPs living in the SPC caused by *Echinococcus multilocularis*, *Taenia martis* and *T. crassiceps* were described [6, 8]. Given the recurrences of these cases, parasitic infections in captive NHPs no longer appear exceptional, and one may wonder to what extent the environment exerts parasite pressure on these animals.

On the other hand, human echinococcosis is not rare in North-Eastern France and seems to have been increasing and spreading in the past 20 years [15, 37, 40]. Furthermore, one of the first described cases of human cysticercosis with *T. martis* was recently reported, following a similar case in a *Macaca tonkeana* subject living in the area [6, 7]. These data show that humans and captive NHPs often share the same environments and are exposed to the same infectious threats. In this particular situation, the SPC is situated close (~100 m) to inhabited areas, notably surrounded by numerous kitchen gardens, crop fields and forest trails frequented by families in a recreational setting.

Thus, the aim of our study was to assess the prevalence of infections by tapeworms belonging to the Taeniidae family (*Taenia* and *Echinococcus* genera) among intermediate and definitive hosts of their wildlife cycle in the close neighborhood of the SPC, as well as to determine the prevalence of these infections in NHPs harbored in the center.

## Materials and methods

### Study population

We studied non-human primates (NHPs) hosted in the SPC, belonging to various species: *Macaca fascicularis*, *Macaca mulatta*, *Macaca tonkeana*, *Chlorocebus aethiops sabaeus*, *Cebus apella*, *Cebus capucinus*, *Eulemur macaco macaco* and *Eulemur fulvus*. These NHPs live semi-freely in parks or large cages. Each park is connected to an indoor facility allowing the animals to shelter. True lemurs and capuchin monkeys are kept indoors during the winter season in order to protect them from cold weather. Cages are divided into two spaces: one inside and the other outside. Monkeys living in parks are fed with dry food, fruits and vegetables but can also access plants and small animals present in the immediate vicinity, whereas monkeys living in cages have only access to distributed food. Monkeys are treated once a year with ivermectin (0.3 mg/kg) and clorsulon (3 mg/kg), alternating with praziquantel (5.7 mg/kg). *Per os* treatment using fenbendazole is administrated with food every year, six months after the praziquantel treatment, without guarantee regarding the effective ingestion of the treatment at the individual level. All NHPs are hosted according to the Directive 2010/63/EU of the European Parliament and of the Council of 22 September 2010 on the protection of animals used for scientific purposes.

### Sample collection

We collected carnivore mammal fecal samples from May 2016 to February 2017. Samples were photographed and GPS coordinates recorded. Samples were stored at 4 °C for up to a week, then at −20 °C. The precise species identification was not established but the belonging to a species of one of the following carnivore species was ensured using an illustrated referential: red fox (*Vulpes vulpes*), Eurasian otter (*Lutra lutra*), least weasel (*Mustela nivalis*), stoat (*Mustela erminea*), European polecat (*Mustela putorius*), European pine marten (*Martes martes*), beech marten (*Martes foina*) and European badger (*Meles meles*). There was no sample collection in NHP parks. Collection was limited to human passage areas.

In parallel, from December 2015 to February 2017, we collected carcasses of rodents found in the center and its neighborhood. Carcasses were discarded when decomposition was too advanced. Every eligible carcass was necropsied. Macroscopic lesions were recorded and tissue integrity was controlled. In every case, a liver sample was collected, in some cases along with kidney, spleen or mesentery samples. All samples were stored at −20 °C.

During necropsy of a young *Macaca fascicularis* of the SPC, carried out for other purposes, a liver sample was collected for analysis despite the absence of lesions suggestive of parasite infection.

We analysed randomly selected sera of animals living in parks, collected during previous annual health control campaigns (2015–2017). During these campaigns, blood collection was performed via femoral vein puncture with a maximum withdrawal of 8.5 mL/kg, after anesthesia using intramuscular ketamine (10 mg/kg), sometimes in combination with dexmedetomidine. Blood was collected in dry tubes and then centrifuged to separate cells and serum. One part of each serum was stored at −80 °C. Selected sera were stored at 4 °C after removal of the serum bank for up to one week before processing. This protocol allowed us to avoid additional manipulations of NHPs otherwise used for ethological studies.

### Molecular analyses

**Figure 3:**
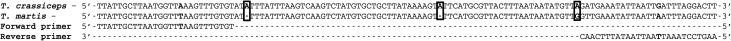
Sequence of the 12S rRNA gene from *T. martis* and *T. crassiceps* amplified by the Tcm-VG primer pair. Differences between the sequences of the two parasites are in bold, and those allowing us to distinguish species are framed.

Polymerase Chain Reaction was used for the detection of *Taenia* spp. and *E. multilocularis*. DNA was extracted from fecal samples using the QIAamp Fast DNA Stool Mini Kit (Qiagen, Netherlands), according to the manufacturer’s instructions. DNA was extracted from tissue samples using the DNeasy Blood & Tissue Kit (Qiagen, Netherlands), according to the manufacturer’s instructions. PCR inhibitors were partly removed using the CHELEX 100 Chelating Ion Exchange Resin (BioRad, USA), according to the manufacturer’s instructions. For *Taenia* spp., we used the JB3/JB4.5 primer pair, formerly designed for *Fasciola hepatica* [18], but also able to amplify a fragment of the *Cox1* mitochondrial gene in other flatworms such as *Taenia* spp. or *Mesocestoides* spp. as previously described [3, 4, 16, 19]. However, given the poor sensitivity of this protocol, partly due to remaining inhibitors, and the difficulty to interpret sequencing results due to lack of specificity of the PCR, we developed a PCR which specifically detects *T. martis* and *T. crassiceps* in fecal samples, using the following primer pair amplifying a 120–122 bp long sequence in the 12S rRNA gene of these parasites: Tcm-VG-F: 5′-TTA TTG CTT AAT GGT TTA AGT TTG TGT-3′; Tcm-VG-R: 5′-AAG TCC TAA ATT AAT TAA TAT TTC AAC-3′. Real-time PCR was carried out on a CFX connect thermocycler (BioRad, USA) in a final volume of 20 μL containing, 10 μL of SsoAdvanced Universal SYBR Green Supermix (BioRad, USA), 2.5 mM MgCl_2_, 0.5 μM of each primer and 2 μL of DNA template. After denaturing at 95 °C for 3 min, 45 cycles were run with 15 s of denaturation at 95 °C, 15 s of annealing at 56 °C, and 30 s of extension at 72 °C. PCR products of all positive samples were subsequently sequenced in order to distinguish *T. martis* and *T. crassiceps*, as described in Figure 3. We observed that the melting peak temperature depended on the species identified: *T. martis* at 75 ± 0.5 °C and *T. crassiceps* at 73.5 ± 0.5 °C, making it possible to distinguish the two species without sequencing. However, in this article, we strictly based our species identification on sequencing. For *E. multilocularis*, we used the EM-H15/EM-H17 primer pair; targeting a sequence of the mitochondrial 12S rRNA gene, as previously described [33]. All PCR products were sequenced by GATC Biotech (Germany). Sequences were compared to the *GenBank* database to determine species.

### Serological analyses

We performed serological analysis on randomly selected NHP blood samples using the ECHINOCOCCUS Western Blot IgG assay (LDBIO Diagnostics, France) for the detection of anti-*Echinococcus* spp. antibodies (in humans, Se = 97.3%, Sp = 95%). We used an indirect immunofluorescence technique for the detection of anti-*Taenia* spp. antibodies using *T. saginata*-derived antigens, according to our laboratory’s internal protocol. This protocol consists in cutting 2 mm wide bands of *T. saginata* proglottid sides, placing these pieces in Cryomount (Histolab, Sweden) or Tissue Tek (Sakura Finetek, Japan) medium before cutting them into 5 μm thick sections using a cryostat. Sections were then placed on slides and fixed in a pure acetone bath for 10 min, then dried for another 10 min, before adding 50 μL of a dilution of the serum to be tested, and incubating for 30 min in a humidity chamber at 37 °C. Slides were then washed using three consecutive PBS baths for 5 min, then one quick distilled water bath before drying. Then, 50 μL of fluorescein marked anti-human antibody solution (Fluoline H, BioMérieux, France) were added to the slide and incubated in a humidity chamber for 30 min, before washing and drying as previously described. Slides were then examined using a fluorescence microscope with a ×10 objective. Each analyzed sample was associated with three controls on the same slide: one negative, one weakly positive, and one strongly positive. Sera were serially diluted twofold, starting at 1/30 initial dilution, until negativity of the test. This technique, which is routinely used for human health purposes in our laboratory, is believed to be suitable for diagnosis in NHPs, since anti-human antibodies are able to react with simian antibodies [6]. In our case, positive controls were diluted serum samples from a confirmed case of peritoneal cysticercosis due to *T. martis* in a *M. tonkeana* showing seroconversion [6]. This test is primarily designed to be used in combination with other, more specific and sensitive, tests. Thus, false positive results might be possible due to cross-reactions with other parasites, in particular other cestodes, or to the presence of rheumatoid factor. All analyses were performed in the certified Laboratory of Parasitology and Medical Mycology of the Strasbourg University Hospital by trained parasitologists and technicians.

### Statistical analyses

Statistical analyses were performed with two-sided tests, with a type I error set at *α* = 0.05. The categorical data are presented as frequency and associated proportions. The differences across groups were compared using the Chi-squared test or Fisher’s exact test, if validity criteria were not met.

## Results

### Fecal sample analysis

**Figure 4:**
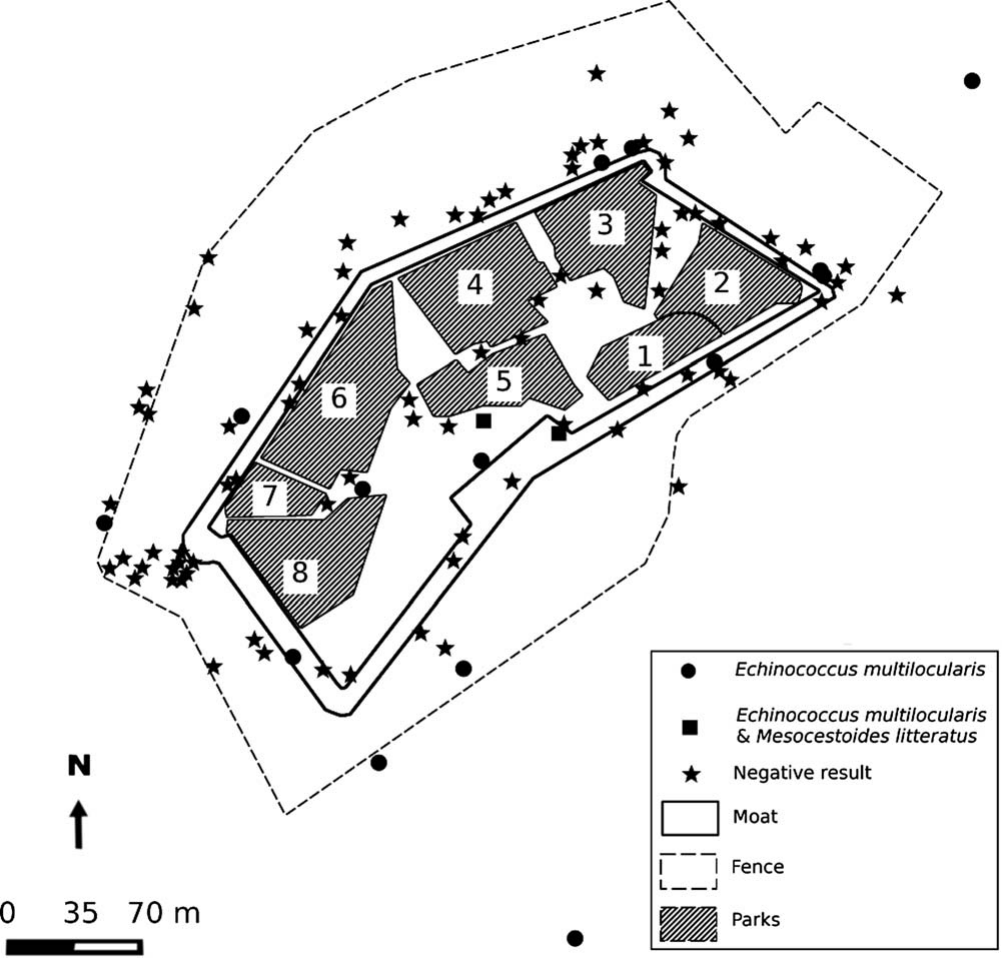
Location of negative and positive carnivore feces for *Taenia* spp. and *Echinococcus* spp.-DNA. Each park is numbered and harbored one species. 1: *Eulemur fulvus*; 2: *Eulemur macaco*; 3: *Cebus capucinus*; 4: *Macaca tonkeana*; 5 & 8: *Macaca mulatta*; 6: *Chlorocebus aethiops*; 7: *Macaca fascicularis*.

One hundred and thirty-eight fecal samples were collected inside the SPC or around the fence surrounding the center (Fig. 4). The search for *E. multilocularis* was positive in 20 samples (14.5%, 95% CI [8.6; 20.4]). Two samples were positive for *Mesocestoides litteratus* (1.5%, 95% CI [0.03; 7.04]). Finally, 7 (5.1%, 95% CI [1.4; 8.7]) fecal samples were found to harbor *T. martis* or *T. crassiceps*. In five samples, we were able to identify the species (four *T. crassiceps*, one *T. martis*). In the two others, the sequencing quality did not allow us to identify the precise species.

### Tissue analysis

Carcasses of twelve small mammals, rodents and insectivores, (two wood mice [*Apodemus sylvaticus*], three black rats [*Rattus rattus*], four common voles [*Microtus arvalis*], one brown rat [*Rattus norvegicus*], one common shrew [*Sorex araneus*], one mouse [*Mus musculus*]) and one cynomolgus monkey were necropsied. None of them presented lesions suggestive of larval cestode infection. Using PCR on liver and kidney tissue extracts, one wood mouse, one common vole and one common shrew were positive for *E. multilocularis* (25%, 95% CI [5.5; 57.2]).

### Serology

We analyzed 61 sera for both *Taenia* spp. and *Echinococcus* spp., and additionally 28 sera for *Echinococcus* spp. only. These individuals were selected randomly, with at least five individuals from each park. All Tonkean macaques were tested. The analysis of 61 NHP sera showed seropositivity for *Taenia* spp. in five individuals (8.2%, 95% CI [1.3; 15.1]). The analysis of 89 NHP sera showed seropositivity for *Echinococcus* spp. in nine individuals (10.1%, 95% CI [3.8; 16.4]). Altogether, 14/90 (15.6%, 95% CI [8.1; 23.1]) tested NHPs were seropositive for, at least, *Echinococcus* spp. or *Taenia* spp. There was no difference in seroprevalences for *Taenia* spp. and *Echinococcus* spp. according to sex (Table 1). Subjects belonging to the genus *Eulemur* showed higher prevalence for *Taenia* spp. than the other species (OR 33.3, 95% CI [3.2; 347.2]). There was no other difference in seroprevalence according to sex or species (Table 2).

**Table 1:** Non Human Primate seroprevalences for *Taenia* spp. and *Echinococcus* spp. according to sex.

	*Taenia* spp.		*Echinococcus* spp.	
	Positive (*n*)	Prevalence (% [95% CI])	Positive (*n*)	Prevalence (% [95% CI])
Female	2/31	6.5 [0.8; 21.4]	5/52	9.6 [3.2; 21.0]
Male	3/30	10 [2.1; 26.5]	4/37	10.8 [3.0; 25.4]
Overall	5/61	8.2 [1.3; 15.1]	9/89	10.1 [3.8; 16.4]

**Table 2:** Non Human Primate seroprevalences for *Taenia* spp. and *Echinococcus* spp. according to species.

Species	Parks	*Taenia* spp.	*Echinococcus* spp.
		Positive (*n*)	Positive (*n*)
*Macaca tonkeana*	4	1/26	5/26
*Macaca mulatta*	5 & 8	0/10	1/15
*Macaca fascicularis*	7	0/5	2/22
*Chlorocebus aethiops*	6	0/5	0/7
*Cebus capucinus*	3	0/5	0/8
*Eulemur macaco*	2	2/5	1/5
*Eulemur fulvus*	1	2/5	0/6

## Discussion

We harvested a substantial number of carnivore mammal fecal samples in the area of the SPC. For all samples, macroscopic characteristics were sufficient to guarantee the origin from a species of carnivore mammals. However, these characteristics did not allow precise species determination, and the difference between mustelids and canids was difficult to assess.

Among all carnivore mammal fecal samples, 14.5% (95% CI [8.6; 20.4]) were positive for *E. multilocularis*-DNA, these samples probably being fox feces since, to our knowledge, there are no documented cases of *E. multilocularis* infection in otters or other mustelids. This figure is similar to what had been observed previously in the Strasbourg area, but far lower than what had already been reported in neighboring Switzerland almost 20 years earlier [10, 14, 21]. Rodents, as prey for carnivores, appeared to present a high prevalence for *E. multilocularis*-DNA, since 25% (95% CI [5.5; 57.2]) of carcasses analyzed were positive. However, this figure should be considered with caution, since only 12 carcasses were analyzed. This prevalence is similar or slightly higher than what had already been described in the Rhine valley [21, 28]. These elements support the existence of a zoonotic cycle of alveolar echinococcosis in the direct environment of the primatology center. Furthermore, 10.1% (95% CI [3.8; 16.4]) of all NHPs were seropositive for *Echinococcus* spp. This prevalence is high compared to the prevalence in humans, around 0.2% [20]. However, it is consistent with the numerous reports of alveolar echinococcosis cases in NHPs living in zoos or primatology centers in Europe and Japan [2, 5, 8, 26, 30, 35]. Given these data, alveolar echinococcosis might be considered an emerging infectious disease in captive NHPs [35]. We found that 1/15 *M. mulatta* subjects and 2/22 *M. fascicularis* subjects were seropositive for *Echinococcus* spp., which can be compared with a previous German study which analyzed an outbreak of infections in *Macaca* spp. causing the death of 12 individuals between 1994 and 2006 [35]. This serological study in apparently healthy monkey’s populations found 12 seropositive subjects, with prevalences of 0.8% in *M. mulatta*, 22.2% in *M. fascicularis*, 7.7% in *M. silenus* and 0.0% in baboons [35]. In this study, seropositive individuals were regularly controlled with ultrasound and remained healthy. Indeed, in these situations, ultrasound screenings might allow early detection of manifest disease [31]. However, given the complete absence of symptoms in seropositive subjects of our study, we did not perform such controls in NHPs primarily used for ethological studies. This situation of high seroprevalence of *Echinococcosis* spp. in NHPs has to be put in parallel with the increase and spreading of human alveolar echinococcosis described in previous studies in the last 20 years, sometimes associated with the increase of red fox populations [14, 15, 32, 37, 40].

In our study, 5/56 (8.2%, 95% CI [1.3; 15.1]) of all tested NHPs were seropositive for *Taenia* spp., only one individual being seropositive for both *Echinococcus* spp. and *Taenia* spp. These results have to be considered with caution as cross-reactions are not excluded with these tests. However, four of these five NHPs were very probably seropositive for taeniids other than *Echinococcus* spp. These results should be viewed in parallel with findings of *Taenia* species in carnivore feces sampled in the surroundings of the SPC with prevalences of 1.5% (95% CI [0.03; 7.04]) for *Mesocestoides* spp.-DNA and 5.1% (95% CI [1.4; 8.7]) for *Taenia* spp.-DNA. These prevalences were lower than what was observed in Switzerland in a previous study, showing prevalences of 4.4% and 16.5%, respectively [21].

Our study has limitations, mainly regarding the diagnostic tools we used. The serological methods are validated for use in humans and could not be strictly validated for our primate population, in the absence of sufficient positive controls. However, concerning *Taenia* spp. serology, it had already been used successfully in a subject with proven *T. martis* infection [6]. Moreover, the detection of the different cestodes in the stool and organs of the final and intermediate hosts was carried out by PCR only, which does not guarantee that positive samples present an infectious risk.

Altogether, to our knowledge, at least 10 different larval cestode infections have been reported in previous studies to affect members of more than 20 different lemur or monkey species (Table 3) [1, 2, 5, 6, 8, 9, 11–13, 17, 22–27, 29, 30, 34–36, 38, 39]. In most of these studies, mortality rates were high, showing that the control of these infections might be of critical importance in maintaining the well-being of NHPs living in parks. Among these parasites, *T. martis*, *E. multilocularis* or *E. granulosus* are three cestodes that may be responsible for human infections. Concerning the source of these infections, a recent study showed that fruits and vegetables used to feed primates harbored in the Zoo of Basel, Switzerland, were frequently contaminated with carnivore’s feces which could be the source of some infections [17]. However, our results on both intermediate and definitive hosts strongly suggest an important role of primate interaction with local wildlife in the development of such infections.

**Table 3:** Larval cestodiasis in NHPs reported in previous studies.

Parasite species	Non-human primate species	References
*Echinococcus multilocularis*	*Macaca mulatta*	[5, 35]
	*Macaca silenus*	[35]
	*Macaca fascicularis*	[1, 8, 13, 31, 35]
	*Macaca sylvanus*	[2]
	*Macaca nigra*	[13]
	*Macaca fuscata*	[25]
	*Lemur catta*	[13, 26, 39]
	*Gorilla gorilla*	[26, 30]
	*Miopithecus talapoin*	[13]
	*Hilobates* spp.	[13]
	*Pongo pygmaeus*	[34]
*Echinococcus granulosus*	*Cercopithecus ascanius*	[2]
	*Macaca nemestrina*	[29]
	*Macaca mulatta*	[24]
*Echinococcus equinus*	*Varecia rubra*	[2, 12]
	*Lemur catta*	[12]
*Echinococcus ortleppi*	*Lemur catta*	[12]
*Echinococcus vogeli*	*Gorilla gorilla*	[22]
*Taenia hydatigena* (cysticercus tenuicollis)	*Macaca fascicularis*	[38]
*Taenia martis*	*Lemur catta*	[11]
	*Macaca tonkeana*	[6]
*Taenia crassiceps*	*Lemur catta*	[27]
	*Eulemur macaco*	Personal data, unpublished
*Mesocestoides* sp.	*Papio cynocephalus anubis*	[23]
	*Saimiri scireus*	[36]
*Spirometra* spp.	*Cercopithecus aethiops*	[9]
	*Papio anubis anubis*	[9]
	*Cercopithecus mitis albortorquas*	[9]

## Conclusions

The description of larval cestode infections in NHPs living in the SPC should draw attention to the potential threat for the human population living near the SPC and consuming potentially contaminated vegetables or using the surrounding hiking paths. Although human infections with *Mesocestoides* spp. are rare, *Echinococcus* spp. and *Taenia* spp. are classical pathogenic parasites for *Homo sapiens*, as is also shown by the increasing incidence of echinococcosis in humans [15, 37, 40], as well as with the first case of human cerebral *T. martis* cysticercosis recently reported, following a similar case in a *M. tonkeana* subject living in the SPC [6, 7]. Since this zoonotic risk is borne by local wildlife, and given the severity of these infections, it seems necessary to implement measures to protect captive NHPs, and to conduct further studies to better assess the risk on human populations.
